# Free posterior tibial artery perforator flap for 2‐stage tracheal reconstruction in patients after resection of well‐differentiated thyroid carcinoma invading the trachea

**DOI:** 10.1002/hed.25675

**Published:** 2019-02-06

**Authors:** Jun Liu, Dan Lu, Di Deng, Ji Wang, Weigang Gan, Jian Zou, Fei Chen, Hui Yang

**Affiliations:** ^1^ Department of Otorhinolaryngology, Head and Neck Surgery West China Hospital, Sichuan University Chengdu China

**Keywords:** 2‐stage, posterior tibial artery perforator flap, thyroid carcinoma, tracheal invasion, tracheal reconstruction

## Abstract

**Background:**

The present study was conducted to explore the efficacy of using a free posterior tibial artery perforator flap (FPTAPF) for trachea reconstruction after resection of well‐differentiated thyroid carcinoma (WDTC) invading the trachea.

**Methods:**

We retrospectively collected and analyzed clinical and surgical data from 14 patients who underwent tracheal reconstruction using a FPTAPF after resection of WDTC invading the trachea between August 2014 and July 2017.

**Results:**

Satisfactory tracheal structure and functional recovery were obtained in 11 of the 14 patients. One patient had breathing difficulties after tracheostomy closure tests because of bilateral recurrent laryngeal nerve damage caused by disease invasion. Tracheostomy incision was not closed in 2 patients because they received postoperative adjuvant radioactive iodine 131 treatment.

**Conclusion:**

Satisfactory tracheal reconstruction results were achieved in patients with resection of WDTC invading the trachea, indicating that a FPTAPF is a safe and reliable choice for management.

## 1 INTRODUCTION

Well‐differentiated thyroid carcinoma (WDTC), including mainly papillary and follicular types, represents the most common endocrine malignancy. WDTC has a good prognosis, with a 10‐year survival rate over 90% after appropriate medical and surgical treatment.^1^ Usually, its therapy options are associated with low morbidity, especially in the patients with early‐stage WDTC.^2^ However, patients with advanced‐stage WDTC often require more aggressive surgery, increasing potential morbidity.

Although laryngotracheal invasion by WDTC occurs less frequently (1%‐13%),^3^ extrathyroid extension and local invasion are associated with an increased risk of tumor recurrence, worsening overall survival prognosis.^4^ Therefore, effective surgical resection of the primary tumor is crucial to the initial management of patients with tracheal invasion by WDTC. However, tracheal reconstruction after tumor resection poses a great challenge for head and neck surgeons. A large number of approaches to tracheal reconstruction have been used, including tracheal end‐to‐end anastomosis,^5^ autologous rib cartilage graft,^6^ composite nasal septal graft,^7^ pedicled sternocleidomastoid clavicular periosteocutaneous flap,^8^ free radial forearm flap combined with biodegradative mesh suspension,^9^ free radial forearm flap combined with titanium mesh or rib cartilage,^10^, ^11^, ^12^ and other free skin grafts.^13^, ^14^ With the development of microsurgical techniques, free skin grafts, such as radial free forearm and free thigh anterolateral flaps, have been widely applied for repairment and reconstruction after head and neck massive surgery.

Several studies^15^, ^16^ indicate that the posterior tibial vessels run along the entire length of the tibia consistently, with a great many septocutaneous perforators regularly clustered in the distal half of the leg. As a result, a free posterior tibial artery perforator flap (FPTAPF) has been considered a good selection in tissue defects repair. In our previous studies,^17^, ^18^ we reported that a FPTAPF could be used in reconstructions of oropharyngeal and hypopharyngeal defects with good prognosis. Therefore, we considered that a FPTAPF might be an attractive alternative approach to tracheal reconstruction after tumor resection for patients with WDTC invading the trachea.

Here, we present our initial clinical experience of using FPTAPF for reconstruction of tracheal defects after excising tumors to treat patients with WDTC invading the trachea, with evaluation of procedure‐related outcomes in these patients.

## 2 PATIENTS AND METHODS

### 2.1 Patients

From August 2014 to July 2017, patients with WDTC invading the trachea were admitted to the Department of Otolaryngology, Head and Neck Surgery, West China Hospital of Sichuan University. Histopathological diagnosis of WDTC was conducted by the Department of Pathology of West China Hospital, Sichuan University. The TNM classification was in accordance with the International Union Against Cancer (2010). All operations were performed by experienced surgeons. All patients received postoperative adjuvant radioactive iodine 131 treatment. The institutional review board of the West China Hospital approved the present retrospective review of medical records.

Patients with an FPTAPF of suitable size and thickness and acceptable recipient vessels and sufficient posterior tibial artery perforators to provide abundant blood supply, which demonstrated by Doppler ultrasound imaging or MRI of lower limb blood vessels with three‐dimensional reconstruction, were considered eligible for this study. Patients who had presented with severe signs and symptoms of peripheral artery disease of the lower limb were excluded.

### 2.2 Operative technique

**Figure 1:**
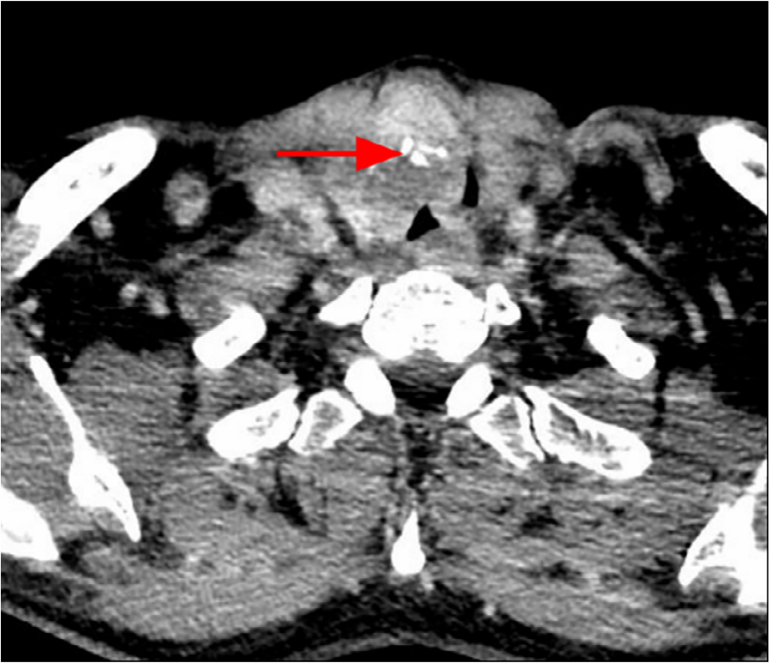
CT showing the extent and size of a tumor. The red arrow indicates thyroid carcinoma invading the trachea

**Figure 2:**
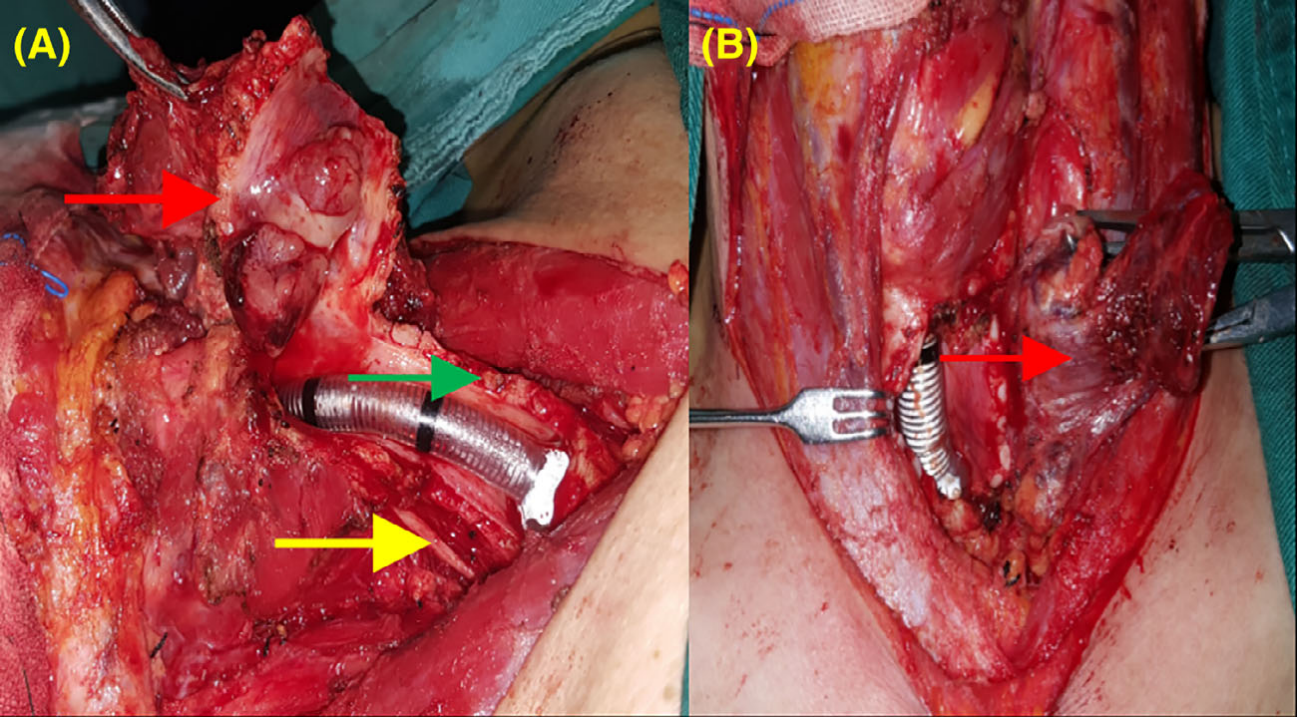
Thyroidectomy and tracheal tumor resection. The yellow arrow indicates the recurrent laryngeal nerve, the red arrow indicates the thyroid and trachea tumor to be removed, and the green arrow indicates the residual tracheal cartilage rings

All patients underwent a preoperative assessment of the lower limb vessels with Doppler ultrasound imaging or MRI of the lower limb blood vessels with 3‐dimensional reconstruction, and CT scans were performed to evaluate the size and extent of their tumor (Figure 1). According to the extent of tracheal stenosis, tracheal intubation was conducted with or without the assistance of fiberoptic bronchoscopy. General anesthesia was then performed for thyroidectomy. During surgery (Figure 2), the parathyroid gland and the recurrent laryngeal nerve were dissected and carefully protected. If the recurrent laryngeal nerve had been encroached by the thyroid tumor, it would be removed except for its segment into the larynx being carefully protected to allow recurrent laryngeal nerve reconstruction. According to the extent of trachea encroachment by WDTC, the tracheal tumor was resected completely. Tumor‐free margins were confirmed by intraoperative frozen‐section pathology results. Intubation was then performed through cervical trachea. Regional neck lymph nodes were dissected and removed in all patients. Available recipient vessels and ansa cervicalis were carefully identified and preserved for later use for microvascular anastomosis and recurrent laryngeal nerve reconstruction, respectively.

**Figure 3:**
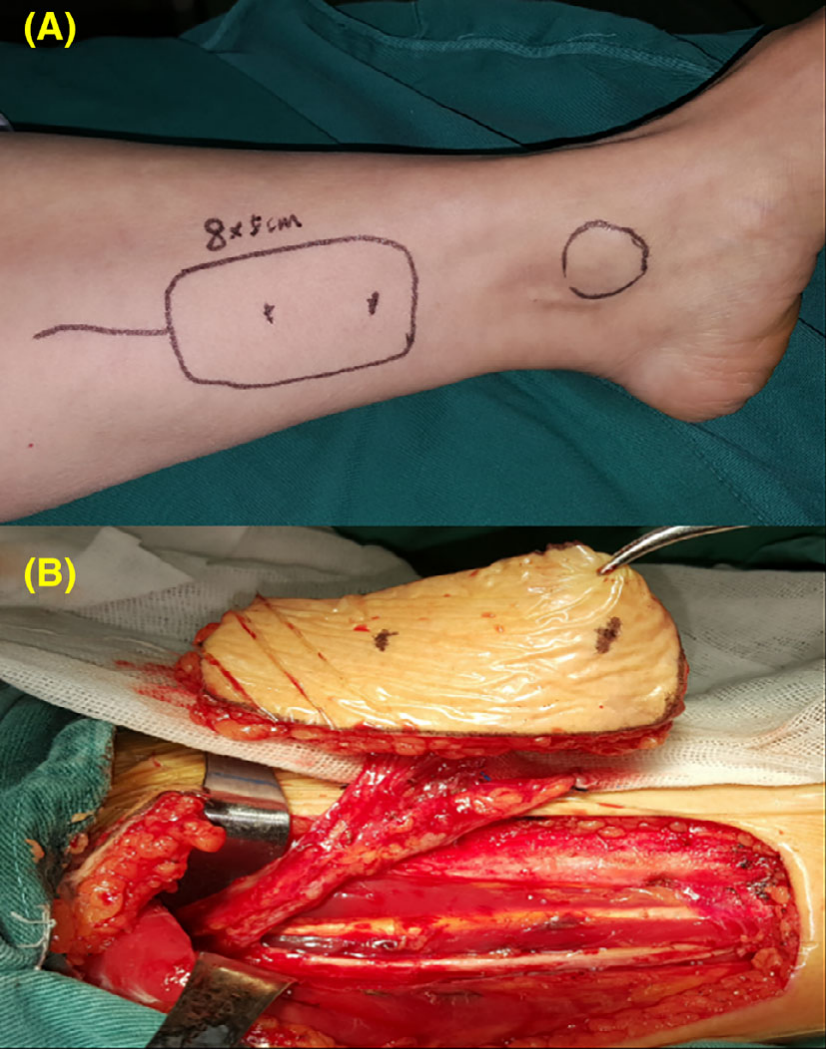
Harvesting of the free posterior tibial flap. A, Design of the posterior tibial flap. B, Harvesting of the posterior tibial flap

**Figure 4:**
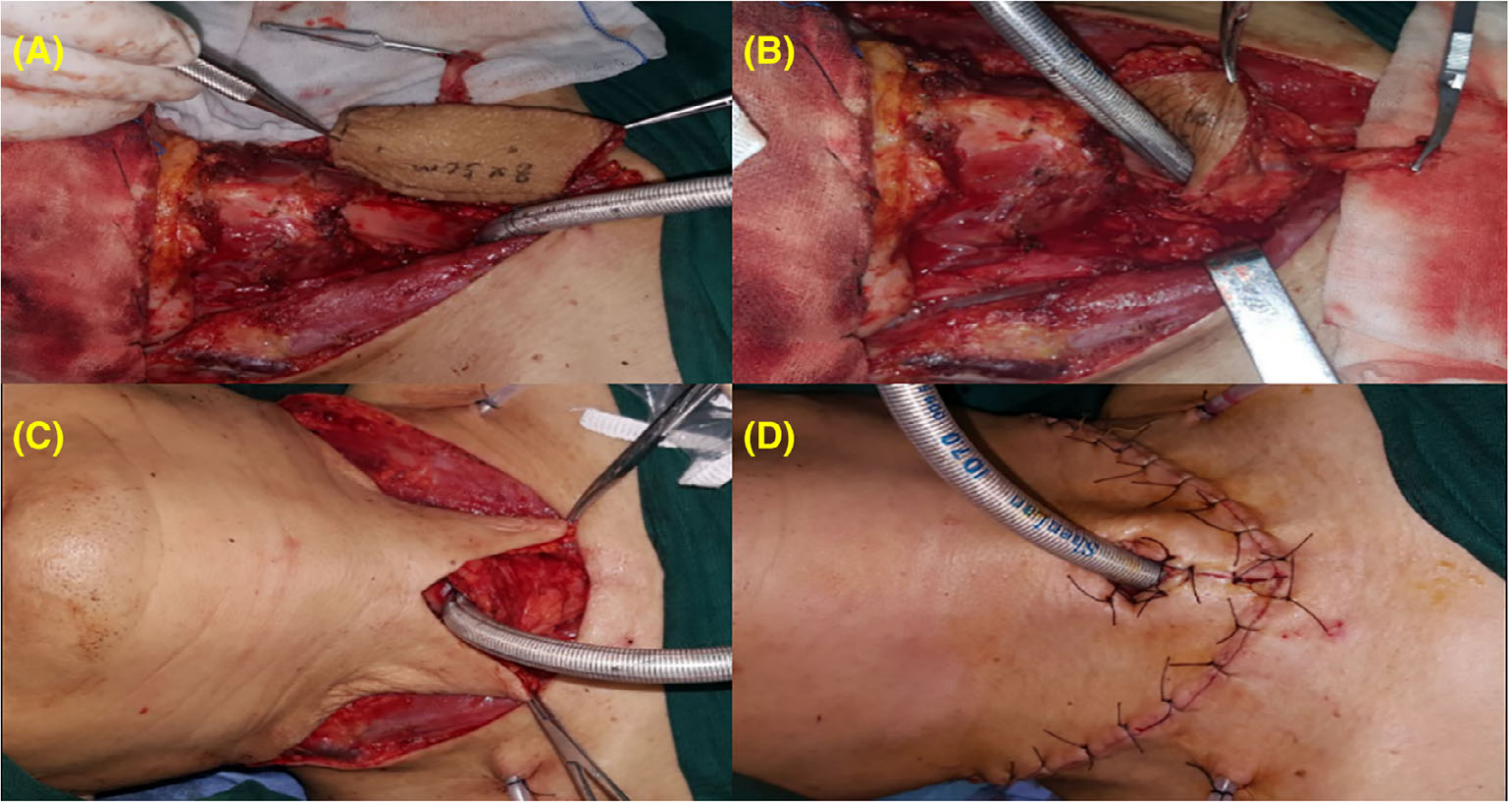
Flap anastomosis. (A, B) The flap was initially sutured to the trachea. (C, D) The flap was sutured to the neck skin as for a permanent tracheostomy

After excision of the tumor, an FPTAPF was harvested (Figure 3). The harvested flap contained 1 greater saphenous vein, 1 posterior tibial artery, and 2 posterior tibial veins, and its actual size was determined by the size of the tracheal defect. Once the recipient vessels were prepared, the posterior tibial artery and veins were transected, and the FPTAPF was transferred to the defect site. As shown in Figure 4, the flap was initially sutured to the trachea (Figure 4A,B), followed by microvascular anastomosis. The posterior tibial artery was anastomosed followed by the posterior tibial veins. The patency of the anastomosed vessels was evaluated by intraoperative microvascular ultrasound, and satisfactory skin temperature and color indicated recovery of the harvested flap. If the recurrent laryngeal nerve was resected partially and the part of the nerve entering the larynx and ansa cervicalis could be identified, the nerve would be reconstructed. The flap was sutured to the neck skin as for a permanent tracheostomy (Figure 4C,D), thus the first‐stage tracheal reconstruction was completed. Skin was grafted onto the leg donor site. Anticoagulant (low molecular weight heparin), spasmolytic, and antibiotic medications were used for all patients postoperatively. Flap inspection of neck surface and through tracheostomal opening was performed every hour to monitor the FPTAPF status, and an electronic laryngoscopy was performed to evaluate the flap thoroughly 3 days postoperatively. After surgical wound recovery, all patients received postoperative adjuvant radioactive iodine 131 treatment.

**Figure 5:**
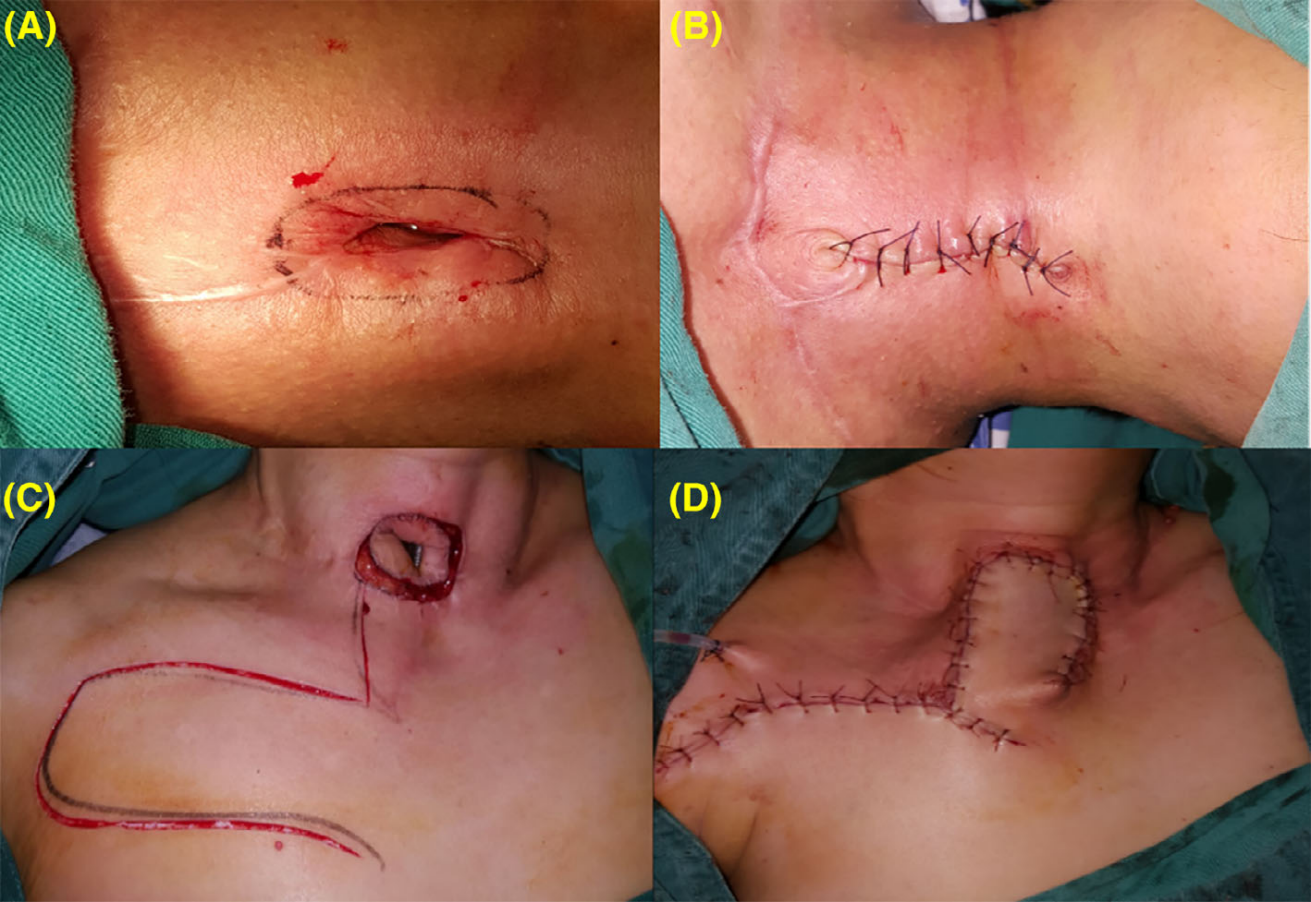
(A, B) The second stage of tracheal reconstruction with a direct suture. (C, D) The second stage of tracheal reconstruction using a local flap

Six months or more postoperatively, a second‐stage tracheal reconstruction was conducted if tracheostomy closure tests showed that the patient had no breathing difficulties. Depending on the condition of the skin around the tracheostomy, direct suture or a local flap was used for the second stage of tracheal reconstruction as shown in Figure 5A,B and Figure 5C,D, respectively.

### 2.3 Outcome evaluation

Data, including age, sex, primary tumor site, tumor pathology, tumor resection range, recurrent laryngeal nerve damage, anastomosed vessels, treatment methods, the size of the FPTAPF, thickness of the FPTAPF, and pedicle length of the FPTAPF, were collected. Primary outcomes were procedure complications, the condition of the reconstructed trachea, breathing function, and patient morbidity during follow‐up.

### 2.4 Statistical analysis

Descriptive statistics were calculated for each study variable. The data are presented as means ± SD. Excel (version 2007 Microsoft, Redmond, Washington ) was used for all statistical analysis.

## 3 RESULTS

Information, including patient age, sex, defect length, and width, whether or not recurrent laryngeal nerve involvement was presented, whether or not recurrent laryngeal nerve reconstruction was performed, flap size, and whether or not rigid support was used, was summarized in Table 1. As shown in this table, of the 14 patients, 10 were female and 4 were male (mean age 53 years, range 30‐75 years). All the patients were diagnosed with thyroid papillary carcinoma using preoperative fine‐needle aspiration pathology with ultrasound guidance. All patients had a tumor of stage T4a (tracheal invasion) and N1b. After resecting the tracheal tumor, tracheal defects ranged from the anterior and unilateral walls of 7 tracheal rings to the anterior, posterior, and unilateral walls of 12 tracheal rings. Of 7 patients with unilateral recurrent laryngeal nerve involvement, 2 underwent unilateral recurrent laryngeal nerve reconstruction. One patient had bilateral recurrent laryngeal nerve involvement and underwent bilateral recurrent laryngeal nerve reconstruction. None of these patients used rigid support including mesh or cartilage.

**Table 1:** Patient and flap data

Patient number	Age	Sex	Defect length	Defect width	RLNI	RLNR	Flap size	Rigid support
1	61	Female	11 tracheal rings	3/4 cir	Unilateral	No	12 cm × 9 cm	None
2	66	Female	14 tracheal rings	1/2 cir	Unilateral	No	12 cm × 7 cm	None
3	65	Female	8 tracheal rings	1/2 cir	Unilateral	No	8 cm × 5 cm	None
4	22	Female	8 tracheal rings	1/2 cir	Unilateral	Yes	8 cm × 5 cm	None
5	20	Female	7 tracheal rings	1/2 cir	No	No	6 cm × 4 cm	None
6	57	Female	8 tracheal rings	1/2 cir	Unilateral	No	8 cm × 5 cm	None
7	75	Female	7 tracheal rings	3/4 cir	Bilateral	Yes	8 cm × 7 cm	None
8	30	Male	7 tracheal rings	1/2 cir	No	No	8 cm × 5.5 cm	None
9	63	Female	7 tracheal rings	1/2 cir	No	No	8 cm × 5 cm	None
10	70	Male	7 tracheal rings	1/2 cir	No	No	6 cm × 5 cm	None
11	52	Female	8 tracheal rings	1/2 cir	Unilateral	No	8 cm × 5 cm	None
12	67	Female	7 tracheal rings	1/2 cir	No	No	8 cm × 5 cm	None
13	28	Male	9 tracheal rings	1/2 cir	Unilateral	Yes	10 cm × 8 cm	None
14	62	Male	9 tracheal rings	1/2 cir	No	No	10 cm × 8 cm	None

Abbreviations: cir, circumference; RLNI, recurrent laryngeal nerve involvement; RLNR, recurrent laryngeal nerve reconstruction.

The sizes of FPTAPFs ranged from 6 cm × 4 cm to 12 cm × 9 cm, the mean thickness was 0.7 cm (range 0.5‐1.2 cm), and the mean flap pedicle length was 10 cm (range 7‐12 cm). The superior thyroid artery and common facial vein were used during the microvascular anastomosis in most patients.

The flap survival rate was 100% and skin grafts at all donor sites healed. During the follow‐up period, no patient experienced cold intolerance of the distal limb, donor site scar pain, or disabilities with regard to leg motion. Overall, 8 patients did very well, without any postoperative complications. However, 4 patients developed postoperative pulmonary infections and 2 patients developed postoperative hypoproteinemia. With anti‐inflammatory agents and nutritional support treatments, all of these postoperative complications were resolved within 2 weeks.

**Figure 6:**
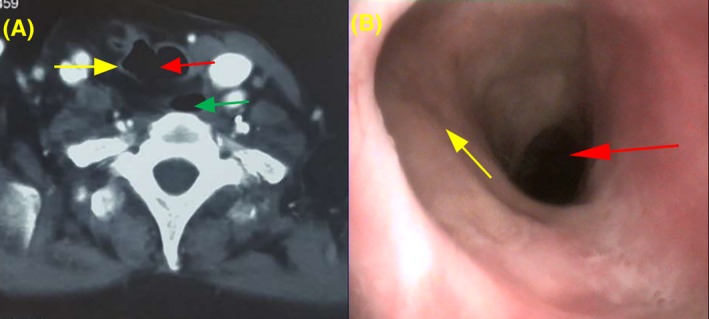
CT scan examination and electronic laryngoscopy of a trachea reconstructed with a FPTAPF. The red arrow indicates the reconstructed trachea, the yellow arrow indicates the FPTAPF, and the green arrow indicates the esophagus

As shown in Table 2, second‐stage tracheal reconstruction was used to restore respiratory and vocal function in 11 patients who had no breathing difficulties after tracheostomy closure tests. Direct suture was used for 8 patients and local flap reconstruction surgery for 3. As shown in Figure 6, CT showed that the average diameter of the reconstructed trachea was 1.1 cm (range 0.8‐1.4 cm) and electronic laryngoscopy showed the reconstructed trachea. However, 1 patient, who had bilateral recurrent laryngeal nerve damage before first‐stage reconstruction, had breathing difficulties after tracheostomy closure tests. Two patients who continued to receive postoperative adjuvant radioactive iodine 131 treatment did not undergo tracheostomy closure. Thus, these 3 patients were tracheostomy dependent.

**Table 2:** Surgical outcomes

Patient	Complications	Decannulation	Second‐stage tracheal reconstruction	Swallow	Speech	Follow‐up
1	None	8 m	Direct suture surgery	Normal	Slightly worse	Well at 40 m
2	PI	7 m	Direct suture surgery	Normal	Slightly worse	Well at 38 m
3	HP	6 m	Local flap reconstruction	Normal	Slightly worse	Well at 37 m
4	None	7 m	Direct suture surgery	Normal	Slightly worse	Well at 35 m
5	None	6 m	Direct suture surgery	Normal	Normal	Well at 31 m
6	None	7 m	Local flap reconstruction	Normal	Slightly worse	Well at 29 m
7	PI	No	NA	Normal	Poor	Well at 20 m
8	None	6 m	Direct suture surgery	Normal	Normal	Well at 18 m
9	PI	6 m	Local flap reconstruction	Normal	Normal	Well at 13 m
10	HP	6 m	Direct suture surgery	Normal	Normal	Well at 11 m
11	None	6 m	Direct suture surgery	Normal	Slightly worse	Well at 10 m
12	PI	7 m	Direct suture surgery	Normal	Slightly worse	Well at 9 m
13	None	No	NA	Normal	Slightly worse	well at 5 m
14	None	No	NA	Normal	Slightly worse	well at 5 m

Abbreviations: PI, pulmonary infection; HP, hypoproteinemia; NA, not available.

The patients were closely followed up for 5‐40 months (Table 2). A thorough test regarding patient's speech and swallowing function was performed after the second‐stage reconstruction. As shown in Table 2, normal swallowing function, indicating the ability to tolerate a regular diet, was reserved in all 14 patients. Normal speech function was reserved in 4 patients, and was only slightly worsened in 9 patients. The aforementioned patient, who had bilateral recurrent laryngeal nerve damage and did not receive second‐stage reconstruction, had a poor speech function outcome. No ischemia event happened at flap donor site.

## 4 DISCUSSION

Management of thyroid cancers with tracheal invasion is a major problem. Shadmehr^19^ reported that complete tracheal tumor resection during thyroidectomy might be curative and associated with improved survival. Therefore, appropriate reconstruction of the trachea following resection of malignancy is of great importance. Head and neck surgeons have used a wide variety of methods^5^, ^6^, ^7^, ^8^, ^9^, ^10^, ^11^, ^12^, ^13^, ^14^ to reconstruct the trachea. However, each method has its own shortcomings.

Long segments of tracheal defects are not suitable for end‐to‐end anastomosis, and the length of trachea defect exceeded 7 tracheal cartilage rings in our present study. Considering the narrow space around tracheal defects, free flaps that are too thick and inconveniently placed pedicle flaps are not suitable for repairing the defects. Therefore, thin free flaps are probably the better choice for reconstruction of long segments of tracheal defects. Maciejewski,^9^ Balasubramanian,^11^ and Thomet^12^ utilized a radial forearm free flap to reconstruct tracheal defects, combined with biodegradative mesh suspension, titanium mesh, and rib cartilage, respectively. They obtained a satisfactory structure and functional repair results. However, there are several disadvantages to the radial forearm free flap. Skin grafting and an unsightly scar on the noticeable surface of the forearm will be unavoidable for most patients.

Compared with a free radial forearm flap, a posterior tibial flap can provide similar tissue properties;^16^, ^17^, ^18^, ^20^ moreover, its unsightly scar has a less noticeable surface than that of a free radial forearm flap. Considering that patients with advanced‐stage WDTC probably need postoperative adjuvant radioactive iodine 131 treatment, and there is a possibility of forming scar adhesions between the FPTAPF and the surrounding tissue after 6 months postoperatively, which may be helpful in reducing airway collapse during inhalation and achieve a better tracheal functional recovery than without scar adhesions, a 2‐stage tracheal reconstruction strategy with a FPTAPF was used to restore normal function of the trachea for WDTC patients in the present study. In the first stage, the FPTAPF was used to construct the bilateral walls of the trachea. A period of 6 months or more were then given to the patients for postoperative adjuvant radioactive iodine 131 treatment and for flap scar adhesions to form. Then a second‐stage tracheal reconstruction was conducted if the conditions of patients permitted. Direct suture or a local flap was used to repair the anterior wall of the trachea in the second stage. Biodegradable mesh suspension, titanium mesh, and rib cartilage were used to replace tracheal cartilage rings as used in previous reconstructions. The lateral wall of the reconstructed trachea should be high enough to achieve a sufficient diameter of the reconstructed trachea to meet the respiratory needs of patients. A beautiful case series reported by Yu et al^10^ demonstrated quite outstanding outcomes when using radial forearm free flap for large tracheal defects with mesh. However, mesh itself being a foreign body and harvesting rib cartilage causing local damage could somehow attribute to adverse outcomes. In the present cases, 1/2 (12 of 14 patients) or 1/4 of cartilage rings (2 of 14 patients) were preserved, partly providing airway support. Meanwhile, the 2‐stage procedure also allowed the flap to have enough time to form scar adhesions, providing a more stable and rigid flap status when the second stage reconstruction was done. Thus, no rigid support was used in our cases. Despite that average diameter of the reconstructed trachea of 1.1 cm (0.8‐1.4cm) was relatively small, long‐term follow‐up also demonstrated a satisfactory tracheal structure and functional recovery, with normal swallowing function reserved in all 14 patients and normal or only slightly comprised in all patients except one with bilateral recurrent laryngeal nerve involvement. Arytenoid resection may be helpful for patients with bilateral recurrent laryngeal nerve involvement to acquire satisfactory respiratory function recovery.

Using FPTAPF, we obtained satisfactory results for tracheal reconstruction. However, tracheal reconstruction with the FPTAPF has several limitations. First, as for the radial forearm free flap, skin grafting and an unsightly scar will be unavoidable in most patients, although perhaps less visibly on the surface of the lower leg. Second, the skill requirement for microvascular anastomosis is high, and the quality of vascular anastomosis is a decisive factor for the success or failure of surgery. Therefore, the promotion of this procedure in primary hospitals might be restricted to the skill level of available surgeons. Third, compared with pedicle skin flaps, FPTAPF requires usable recipient vessels from the neck. Obviously, if reliable neck recipient vessels cannot be found to conduct the microvascular anastomosis, free flaps cannot be used to reconstruct the trachea. Fourth, a major tributary of the lower limb is lost, which could endanger the circulation and subsequently lead to ischemia. Therefore, strict inclusion criteria must be used to exclude those patients with insufficient distal limb circulation. No ischemia event was noted during our follow‐up, which might indicate that strict patient inclusion and careful surgery performance might be able to concur the possibility of ischemia brought by tributary compromise. Finally, even though neither breathing nor speech function was compromised by the slightly smaller diameter of reconstructed trachea during our long‐term follow‐up except for only 1 patient with bilateral recurrent laryngeal nerve involvement, there might be a possibility of flap collapse in the future. And this possibility might could be addressed by rigid. However, considering our quite acceptable outcomes and possible adverse outcomes brought by rigid support, it might be concluded that our 2‐stage reconstruction could be considered in selected patients with WDTC. Further studies might be needed to investigate efficacy of different flaps and absence/presence of rigid support for massive neck reconstruction.

We note that avoiding pressure on the skin flap and observing closely the blood flow in grafted flaps after the operation is very important and necessary to detect potential skin flap crises early so that timely and appropriate interventions can be made to save the skin flap. Adequate preoperative preparation, strict inclusion criteria, complete tumor resection, an appropriate tracheal reconstruction strategy, and reasonable postoperative management should guarantee that a patient obtains ideal tracheal structure and function after tumor resection and tracheal reconstruction.

## CONSENT

Written informed consent was obtained from all patients for presentation of the paper and accompanying images.

## CONFLICT OF INTEREST

None declared.
